# Frequency-Switchable Microfluidic CSRR-Loaded QMSIW Band-Pass Filter Using a Liquid Metal Alloy

**DOI:** 10.3390/s17040699

**Published:** 2017-03-28

**Authors:** Seunghyun Eom, Muhammad Usman Memon, Sungjoon Lim

**Affiliations:** School of Electrical and Electronics Engineering, College of Engineering, Chung-Ang University, 84 Heukseok-ro, Dongjak-gu, Seoul 156-756, Korea; umsh0303@gmail.com (S.E.); musmanm@outlook.com (M.U.M.)

**Keywords:** band-pass filter, QMSIW, 3D printing, microfluidic channel, CSRR, EGaIn, frequency-switchable

## Abstract

In this paper, we have proposed a frequency-switchable complementary split-ring resonator (CSRR)-loaded quarter-mode substrate-integrated-waveguide (QMSIW) band-pass filter. For frequency switching, a microfluidic channel and liquid metal are used. The liquid metal used is eutectic gallium-indium (EGaIn), consisting of 24.5% indium and 75.5% gallium. The microfluidic channels are built using the elastomer polydimethylsiloxane (PDMS) and three-dimensional-printed microfluidic channel frames. The CSRR-loaded QMSIW band-pass filter is designed to have two states. Before the injection of the liquid metal, the measured center frequency and fractional bandwidths are 2.205 GHz and 6.80%, respectively. After injection, the center frequency shifts from 2.205 GHz to 2.56 GHz. Although the coupling coefficient is practically unchanged, the fractional bandwidth changes from 6.8% to 9.38%, as the CSRR shape changes and the external quality factor decreases. After the removal of the liquid metal, the measured values are similar to the values recorded before the liquid metal was injected. The repeatability of the frequency-switchable mechanism is, therefore, verified.

## 1. Introduction

Improvements in frequency-switchable devices, such as PIN diodes [1,2,3,4], varactor diodes [5,6,7,8], radio frequency microelectromechanical systems (RF MEMS) [9,10,11,12], and field-effect-transistor (FET) switches [13,14] have enabled the growth of frequency-switchable technology, owing to its reliability and versatility. However, these switchable mechanisms need additional DC bias networks. Isolating the biasing and control circuits can be a challenging task. 

Microfluidic technologies are emerging as one of the alternative switching mechanisms because these methods do not require additional DC bias networks [15]. A conduction path can be established by filling the microfluidic channels with a conductive material [16,17,18]. In the case of frequency-switchable applications, to fill microchannels with conducting materials, a liquid metal, such as eutectic gallium-indium (EGaIn, indium (In) 24.5% and gallium (Ga) 75.5%) is extensively used, since it is easy to inject and non-toxic in nature [19,20,21]. Injecting the liquid metal inside microfluidic channels constructed over an elastomeric substrate (e.g., polydimethylsiloxane (PDMS)) has proven to be an easier technique for the fabrication of microwave antennas and circuits and making them reconfigurable. For example, dipole antennas have been designed by using liquid metal [22]. In addition, ground planes were fabricated by using liquid metal-filled meshed microfluidic paths [23]. Unlike conventional microwave structures which are made of solid metal elements, these fluidic RF structures incorporate the mechanical properties of the sheathing material (e.g., PDMS); therefore, they are flexible and mechanically durable. Injecting liquid metal into microfluidic channels offers a simple method for shaping the metal into useful structures such as switches, to achieve reconfigurability in filters, antennas, and other similar devices. Since the liquid metal is a low-viscosity liquid, these structures adapt the mechanical properties of the PDMS microchannels. Consequently, the structures return to their original shape after being deformed. Mercury, a well-known liquid metal, cannot be used in microfluidics because it has the tendency to lead up to minimize its surface energy and, hence, draws out unexpectedly from the microchannels. In contrast to mercury, EGaIn forms a thin oxide skin which provides a mechanically stable formation of the liquid after it is inserted into the microchannels [24].

The substrate-integrated-waveguide (SIW) cavity resonator is a well-known technology that provides advantages of compact size, easy fabrication, and low leakage loss [25,26]. In particular, we introduced a quarter mode substrate-integrated-waveguide (QMSIW) cavity resonator, with approximately 75% reduction in size compared to a full SIW resonator, while maintaining the same resonant frequency [27,28,29]. Previously, the microfluidic eight-mode SIW (EMSIW) resonator was proposed, where the microfluidic channel was loaded on the open sides of the EMSIW for reconfigurable antenna applications [30]. Although an EMSIW resonator is more compact than a QMSIW resonator, it is not a good candidate for a filter application owing to the high radiation loss.

In this study, we propose a frequency-switchable CSRR-loaded QMSIW band-pass filter. A complementary split-ring resonator (CSRR) is a squared-slot structure with the split on the metallic pattern. When the CSRR is loaded on the top plane of the QMSIW structure, we can achieve a further size reduction without requiring additional space. For the frequency-switchable feature, we utilized a microfluidic channel using a polydimethylsiloxane (PDMS) substrate with EGaIn. The microfluidic channel was fabricated using a three-dimensional (3D) printer which provided simpler and faster fabrication compared to conventional lithography [31,32].

## 2. CSRR-Loaded QMSIW Band-Pass Filter Design 

For designing the proposed band-pass filter, the finite-element-method (FEM)-based ANSYS high-frequency structure simulator (HFSS) was used. It has also been used in a previous study on band-pass filter design [33].

The equation used to determine the resonant frequency of the rectangular SIW cavity resonator is [34]:

(1) $$f_{mn0}=\frac{1}{2\pi \sqrt{\mu \varepsilon }}\sqrt{{\left(\frac{m\pi }{W_{SIW}}\right)}^{2}+{\left(\frac{n\pi }{L_{SIW}}\right)}^{2}}$$

where *ε* and *μ* represent the permittivity and permeability of the dielectric material inside the cavity, respectively. *L_SIW_* and *W_SIW_* are the length and the width of the SIW, respectively, while m and n are the mode numbers. The lowest resonant frequency corresponds to the TE_100_ mode.

**Figure 1:**
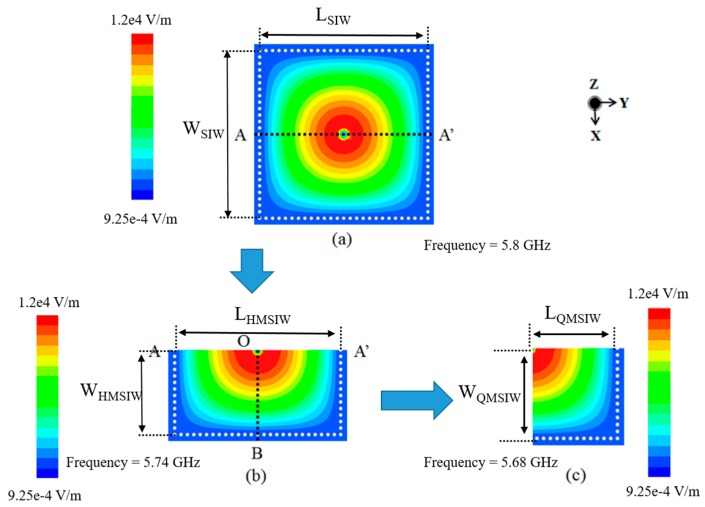
The simulated magnitude of E-field distributions for (**a**) full-mode SIW; (**b**) half-mode SIW; and (**c**) quarter-mode SIW at their dominant resonant frequencies.

Figure 1 shows the simulated magnitude of the electric field distributions of the full SIW, half-mode SIW (HMSIW), and QMSIW resonators. When the SIW cavity resonator is cut on the AA’ plane along the magnetic wall, an HMSIW resonator can be realized [35,36]. Similarly, the QMSIW resonator can be realized by cutting on the OB plane of the HMSIW [37,38].

Ideally, the QMSIW is half that of the HMSIW size. However, fringe fields are generated from the open sides along *W_QMSIW_* and *L_QMSIW_*. Δ*L_QMSIW_* and Δ*W_QMSIW_* are the effects of length and width extensions owing to fringe fields, respectively. Therefore, the resonant frequency of the QMSIW cavity at the TE_100_ dominant mode is given by:

(2) $$f_{100,QMSIW}=\frac{1}{2\pi \sqrt{\mu \varepsilon }}\sqrt{{\left(\frac{\pi }{W_{QMSIW}+\Delta W}\right)}^{2}+{\left(\frac{\pi }{L_{QMSIW}+\Delta L}\right)}^{2}}$$

The magnitude of the E-field distribution of the QMSIW is kept similar to that of the full-mode SIW, as shown in Figure 1. Thus, the QMSIW cavity resonator is 1/4 the size of the SIW, while maintaining almost the same resonant frequency as that of the SIW. In this study, the QMSIW resonator is employed to design a compact filter.

**Figure 2:**
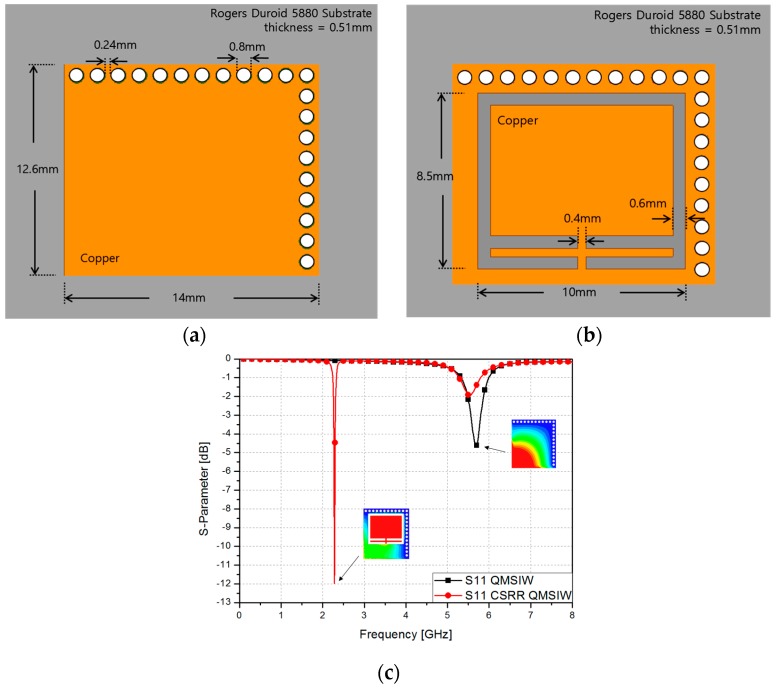
Geometry of (**a**) QMSIW and (**b**) CSRR-loaded QMSIW; (**c**) simulated S-parameters of CSRR-loaded QMSIW and QMSIW with the E-field distribution layout.

Figure 2a shows the geometry and dimensions of a single QMSIW cavity, while Figure 2b shows the same QMSIW cavity with a CSRR installed over it. It can be seen that the structure is planar, the gray part is the dielectric material, which is a Rogers Duroid 5880 substrate having a thickness of 0.51 mm. The orange part is copper and the white part is the integrated gold vias connecting the top and bottom parts of the structure. As illustrated in Figure 2c, the resonant frequency of the CSRR-loaded QMSIW is reduced from 5.68 to 2.28 GHz. This represents a 59.86% reduction. As observed, the electric field is confined around the CSRR rather than at the edges of the open sides of the QMSIW. The CSRR interacts with the QMSIW structure to create a new propagation mode [29]. The physical size of the CSRR-loaded QMSIW is 14 mm × 24 mm. Its electrical length is 0.1188*λ_g_* × 0.1283*λ_g_*, which is calculated at 2.28 GHz.

**Figure 3:**
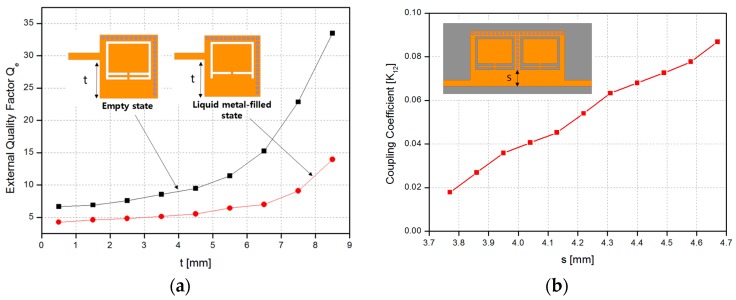
(**a**) External quality factor variation with distance *t* for the CSRR-loaded QMSIW resonator. (**b**) The coupling coefficient of coupled cavities with distance *s*.

The external quality factor (*Q_e_*) can be controlled by the offset distance *t* from the feeding line, as shown in Figure 3a. The coupling coefficient K_12_ can be controlled by the offset distance s, as shown in Figure 3b. The coupling coefficient can be calculated using the following coupling coefficient method [39]:

(3) $$K_{1,2}=\frac{{f_{p2}}^{2}-{f_{p1}}^{2}}{{f_{p2}}^{2}+{f_{p1}}^{2}}$$

where *f_p_*_1_ and *f_p_*_2_ are the two split resonant frequencies of a pair of coupled resonators. The band-pass filter design has been studied in many previously reported studies [28,29,36]. The CSRR-loaded QMSIW is designed using the *n*-order Chebyshev low-pass prototype value. The coupling coefficient and external quality factor can be determined by using the following equation:

(4) $$K_{i,i+1}{|}_{i=1\;to\;n-1}=\frac{FBW}{\sqrt{g_{i}g_{i+1}}}\;Q_{e}=\frac{g_{0}g_{1}}{FBW}$$

where *g* and *FBW* are the Chebyshev low-pass filter prototype value and fractional bandwidth, respectively.

**Figure 4:**
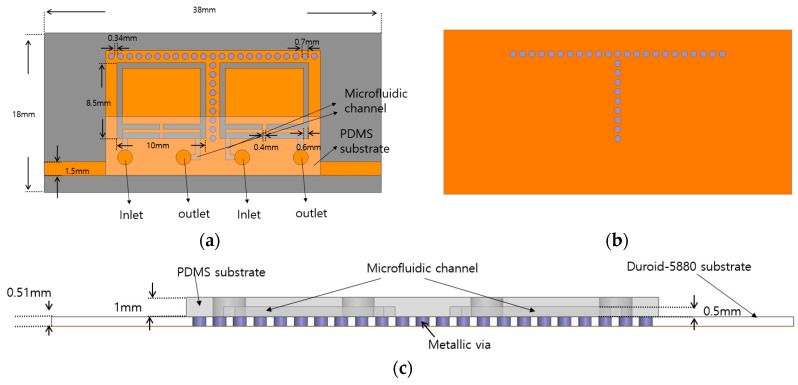
(**a**) Top view of the CSRR-loaded QMSIW filter with a PDMS substrate; (**b**) bottom view of the proposed filter; and (**c**) side view of the proposed filter.

Figure 4 shows the layout of the proposed CSRR-loaded QMSIW band-pass filter, which is composed of two different substrates. The relative permittivity, thickness, and dielectric loss of the Duroid-5880 substrate are 2.2, 0.51 mm, and 0.0009, respectively. The PDMS substrate is attached on top of the Duroid-5880 substrate to form the microfluidic channels. The relative permittivity, thickness, and dielectric loss of PDMS are 2.8, 1 mm, and 0.02, respectively. 

**Figure 5:**
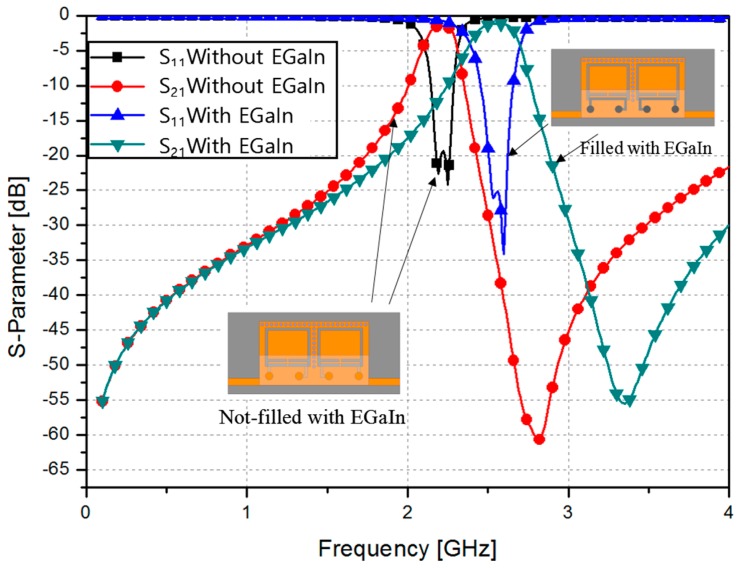
Simulated S-parameter of the CSRR-loaded QMSIW before and after injecting EGaIn.

**Figure 6:**
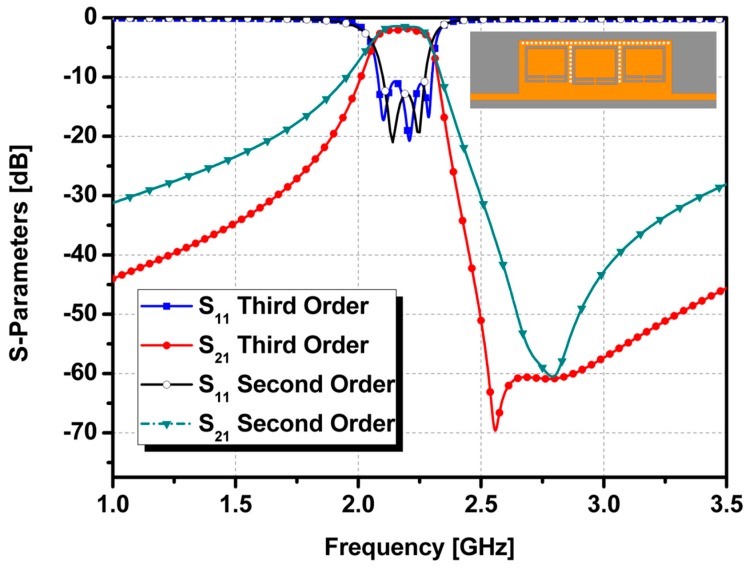
Designs of second-order and third-order CSRR-loaded QMSIW band-pass filters and their simulated S-parameter results.

A second-order band-pass filter was implemented using CSRR-loaded QMSIW cavities with a center frequency of 2.205 GHz, fractional bandwidth of 7.71%, and two transmission zeros at 2.19 GHz and 2.25 GHz. An insertion loss of less than 1.65 dB with an in-band and a return loss greater than 19 dB were achieved. Figure 5 shows the simulated S-parameter results when the microfluidic channels are empty and filled with liquid metal. After injecting the liquid metal, the center frequency shifted from 2.205 GHz to 2.545 GHz. An insertion loss of less than 1.3 dB with an in-band and a return loss greater than 24 dB were achieved. Although the coupling coefficient was practically the same, the fractional bandwidth changed from 7.17% to 10.61% because of the change in the CSRR shape and a decrease in the external quality factor, as shown in Figure 3a. To demonstrate the reliability of the idea, a third-order band-pass filter was also designed using the same mechanism. The design of the third-order bandpass filter and its frequency response are shown in Figure 6.

**Figure 7:**
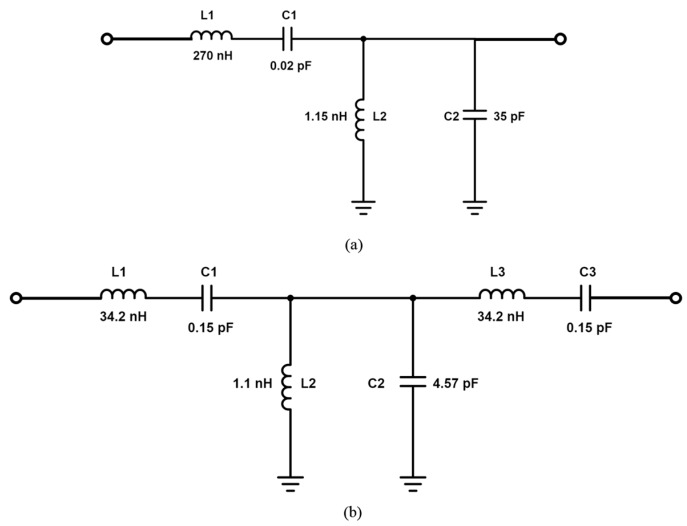
Equivalent circuits of (**a**) proposed second-order band-pass filter design and (**b**) third-order bandpass filter design shown in Figure 6.

In Figure 6, the S-parameter results of second-order and third-order CSRR-loaded QMSIW band-pass filters are compared. Thus, the proposed filter design can be extended to higher orders by using the demonstrated approach. The equivalent circuits for the proposed second-order and demonstrated third-order bandpass filter designs are shown in Figure 7.

## 3. Fabrication and Measurement

**Figure 8:**
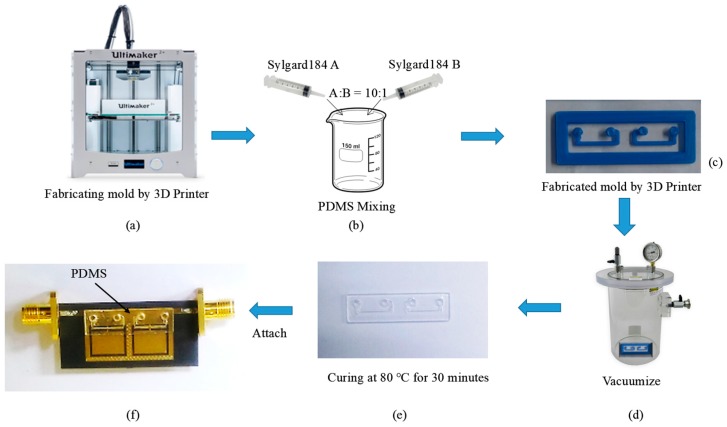
Fabrication process of the microfluidic channel: (**a**) Fabricating the mold using a 3D printer; (**b**) mixing of the PDMS solution; (**c**) pouring of the PDMS solution into the mold/frame; (**d**) vacuuming; (**e**) curing; and (**f**) Final sample (PDMS attached to the Duroid substrate).

Figure 8 shows the fabrication process of the PDMS substrate with the microfluidic channel. As shown in Figure 8a, a mold of the microfluidic channel was initially fabricated by using a 3D printer (Ultimaker 2, Geldermalsen, The Netherlands). After considering the printing resolution, a mold with a thickness of 0.5 mm was built by using the 3D printer, which became the thickness of the microfluidic channel where the liquid metal would be sustained. Further, as shown in Figure 8b, a PDMS silicone elastomer base (Sylgard184 A) and a curing agent (Sylgard184 B) were mixed in a ratio of 10:1. A vacuum pump was then used to remove unnecessary air bubbles from the mixed solution. To make a PDMS substrate, the uncured solution was poured into the mold of the microfluidic channel as shown in Figure 8c. It was cured at 80 °C for 30 min as shown in Figure 8d,e. Finally, the PDMS with the microfluidic channel was obtained. 

The QMSIW CSRR pattern was realized by using a conventional PCB manufacturing process on a 0.51-mm-thick Duroid-5880 substrate. To attach the Duroid-5880 and PDMS substrates, the uncured PDMS solution was used as an adhesive. Figure 8f shows the final sample, where PDMS is attached at the position indicated (high E-field position of the CSRR).

**Figure 9:**
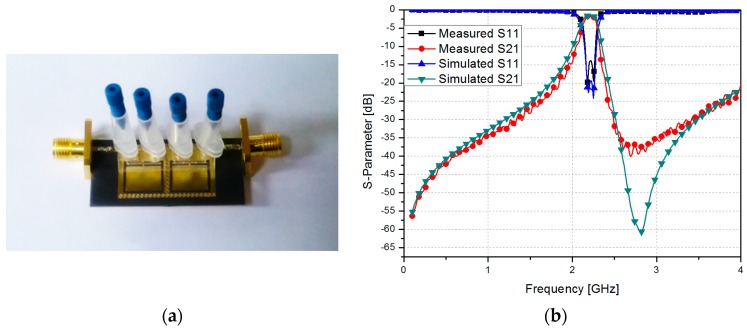
(**a**) Fabricated CSRR-loaded QMSIW filter prototype and (**b**) simulated and measured S-parameter of the proposed CSRR-loaded QMSIW filter.

Figure 9a shows the fabricated QMSIW CSRR filter prototype. The return loss and insertion loss of the filter were recorded using an Anritsu MS2038C vector network analyzer. Figure 9b shows the simulated and measured S-Parameters of the proposed filter along with their comparison. The measured center frequency and fractional bandwidths were 2.205 GHz and 6.80%, respectively. Two transmission zeros were measured at 2.18 GHz and 2.25 GHz. An insertion loss of less than 1.8 dB with an in-band and a return loss greater than 14 dB were achieved.

**Figure 10:**
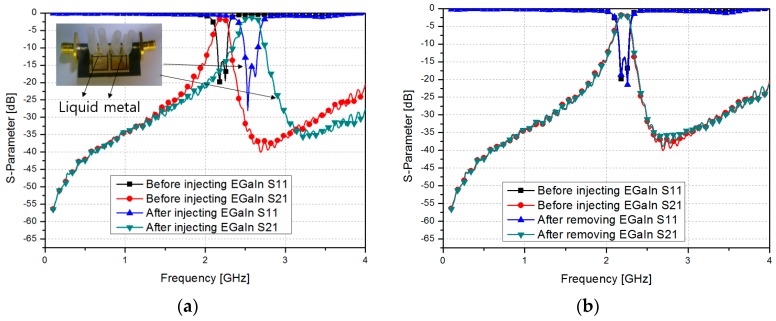
Measured S-parameter of the fabricated CSRR-loaded QMSIW (**a**) before and after injecting the microfluidic channels with EGaIn; and (**b**) before injecting EGaIn and after the removal of EGaIn.

Figure 10a shows the measured S-parameter results before and after the microfluidic channels were filled with liquid metal. The liquid metal was injected using a micropipette. After the injection, the center frequency shifted from 2.205 GHz to 2.56 GHz. An insertion loss of less than 1.5 dB with an in-band and a return loss greater than 15 dB were achieved. The fractional bandwidth changed from 6.8% to 9.38%. 

After the removal of the liquid metal, a small amount of liquid metal remained on the PDMS surface. However, it did not come in contact with the CSRR directly. Therefore, as shown in Figure 10b, the measured S-parameter results before and after injecting the liquid metal are practically the same.

## 4. Conclusions

In this research, we proposed a frequency-switchable CSRR-loaded QMSIW band-pass filter. Microfluidic channels were built using the PDMS elastomer and microfluidic channel frames. A 3D printer was used for the fabrication of the microfluidic channel frames. The size of the proposed band-pass filter is 0.2376*λ_g_* × 0.1283*λ_g_*, where *λ_g_* is the guided wavelength at 2.28 GHz. The CSRR-loaded QMSIW band-pass filter is designed to have two states. Before injecting the liquid metal, the measured center frequency and fractional bandwidths were 2.205 GHz and 6.80%, respectively. An insertion loss of less than 1.8 dB with an in-band and a return loss greater than 14 dB were achieved. After injecting the liquid metal, the center frequency shifted from 2.205 GHz to 2.56 GHz. An insertion loss of less than 1.5 dB with an in-band and a return loss greater than 15 dB were achieved. Although the coupling coefficient was practically the same, the fractional bandwidth changed from 6.8% to 9.38% as the CSRR shape changed and the external quality factor decreased. After the removal of the liquid metal, the measured results were practically the same as those before injection. Consequently, the repeatability of the frequency-switchable mechanism was verified.
